# Spatio-Temporal Feature Enhancement for Recognizing Strongly Correlated Sequential Actions in Aircraft Assembly

**DOI:** 10.3390/s26123781

**Published:** 2026-06-13

**Authors:** Jiaming Shi, Xiang Huang, Guoyi Hou, Chengda Guo, Qingxue Wang, Yumin Chen

**Affiliations:** College of Mechanical and Electrical Engineering, Nanjing University of Aeronautics and Astronautics, Nanjing 210016, China; jiamingshi@nuaa.edu.cn (J.S.); nuaa_hx@nuaa.edu.cn (X.H.); nuaa_gcd@nuaa.edu.cn (C.G.); wangqingxue@nuaa.edu.cn (Q.W.); logym@nuaa.edu.cn (Y.C.)

**Keywords:** aircraft assembly, action recognition, long-term sequential actions, computer vision, wing assembly process

## Abstract

The positioning and clamping process in aircraft assembly exhibits pronounced long-term temporal correlations and intense human–machine interactions. Consequently, assembly quality depends heavily on operator compliance and consistency. Capturing long-term, strongly correlated features in complex industrial environments remains a significant challenge. To overcome this, this study proposes a Long-Term Strongly Associated Action Recognition Network (LTSA-Net) tailored for aircraft assembly positioning and clamping tasks. Based on the C3D backbone, the model first incorporates the SimAM attention mechanism and BN modules to significantly enhance focus on critical spatiotemporal features. To address the challenge of capturing long-term temporal dependencies, LTSFEM is designed to extract global temporal information accurately. Furthermore, to balance structural lightweight design with real-time inference requirements, the CWSTB module is integrated to achieve substantial parameter compression. In addition, a dedicated aircraft assembly positioning and clamping dataset was constructed, and a robust training framework was established using the AdamW optimizer and Mixup data augmentation. Experimental results demonstrate that LTSA-Net achieves a recognition accuracy of 98.82% on the LTSA-Dataset, with a per-frame inference time of 42 ms, successfully meeting the dual requirements of high precision and real-time performance in industrial scenarios, and providing a practical technical solution for intelligent monitoring of aircraft assembly processes.

## 1. Introduction

As a core stage in aerospace manufacturing, aircraft assembly directly dictates the aerodynamic performance and operational safety of the final vehicle. The Industry 4.0 paradigm has significantly accelerated the adoption of automation technologies. However, automated systems still struggle to balance high efficiency with execution flexibility when handling high-precision, low-stiffness, and large-scale structural components. Consequently, manual assembly remains indispensable, accounting for over 60% of critical assembly operations due to its superior cognitive flexibility and environmental adaptability. This human-centered paradigm effectively navigates the complex structural topologies of aircraft assembly and will remain a foundational pillar for the foreseeable future.

Within this human-centered aircraft assembly paradigm, positioning, clamping, and joining constitute the three fundamental pillars of the assembly process. Taking the assembly of a typical wing box as an example, the positioning and clamping processes of core components such as panels, spars, and ribs exhibit pronounced long-duration characteristics and a high density of fine-grained actions. Although individual operations present relatively low technical difficulty, this stage is a crucial prerequisite for subsequent joining. Consequently, it represents a critical node in assembly quality control and a vulnerable stage highly susceptible to human-induced uncertainties. Due to the heavy reliance on operator experience and attentiveness, this study identifies three representative categories of behavioral anomalies occurring during the positioning and clamping stage:

- Process omission: During the positioning and clamping of individual components, the omission of critical operations, such as the positioning and alignment of key components, directly compromises the integrity of subsequent joining processes.
- Non-compliant operation: Operations deviating from prescribed workflows often rely on personal experience or excessive force to adjust low-stiffness components. This practice leads to unintended elastic deformation and residual internal stresses, which severely degrade the accuracy of the final aerodynamic profile.
- Fatigue-induced errors: Constrained by narrow and enclosed working environments, prolonged operations are prone to inducing both physical and cognitive fatigue, which in turn leads to positioning deviations or inadvertent operational errors.

**Figure 1:**
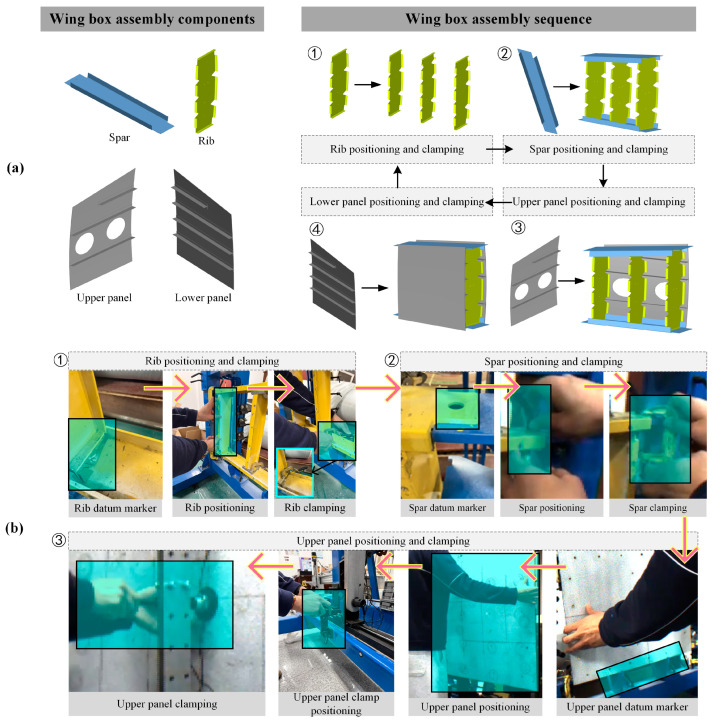
Overview of the positioning and clamping workflow in wing box assembly, (**a**) provides a detailed illustration of the key components involved in wing box assembly and the overall operational workflow, while (**b**) focuses on the specific procedural steps of the positioning and clamping process. It should be noted that, since the positioning and clamping procedures for the lower and upper skins are identical in terms of operational logic, only the workflow for the upper skin is presented in the figure to avoid redundancy.

To address the aforementioned challenges, this paper proposes an intelligent monitoring system based on computer vision techniques, which enables real-time detection and proactive intervention of process omissions, non-compliant operations, and fatigue-induced errors through in-depth analysis of visual data captured in the assembly environment. As illustrated in Figure 1, the positioning and clamping procedures of panels, spars, and ribs in the wing box assembly are abstracted into a hierarchical behavioral sequence. Unlike conventional short-duration video classification tasks, the positioning and clamping process in aircraft assembly exhibits pronounced untrimmed and continuous characteristics. Considering the large number of fine-grained sub-actions involved and their strong contextual dependencies, this study models such assembly actions as long-term, strongly correlated actions.

With the rapid advancement of computer hardware, software, and multimedia technologies, human action recognition has emerged as a critical research area within the field of computer vision [1]. By analyzing video streams to extract salient visual cues, this technology enables computer systems to emulate human perceptual mechanisms, thereby achieving autonomous perception and adaptation to operational environments. However, the distinctive characteristics of industrial environments pose three significant challenges for action recognition in positioning and clamping operations:

- Long-duration continuity: Action samples span extended temporal ranges, within which informative action segments are often sparsely distributed across long background streams, making the extraction of discriminative features particularly challenging.
- Strict temporal–causal constraints: Action sequences are not independent but follow a rigid process-topology order with strong contextual dependencies, rendering single-frame recognition insufficient in terms of semantic understanding.
- Fine-grained spatiotemporal dynamics: Unlike actions characterized by large-scale body movements, positioning, and clamping involve numerous subtle hand manipulations and tool interactions, requiring models to capture fine-grained spatiotemporal evolutions under complex background conditions.

To overcome the aforementioned limitations, this paper proposes a long-term, strongly correlated action recognition approach for positioning and clamping operations in aircraft assembly. By systematically comparing mainstream action recognition methods, an effective recognition model is constructed, and an action-supervision feedback mechanism is established, thereby improving the quality of positioning and clamping assembly and providing a new pathway toward intelligent aircraft assembly. The contributions of this work are summarized as follows:

- An enhanced spatiotemporal feature extraction network, termed LTSA-Net, is proposed. By integrating 3D SimAM attention and batch normalization (BN) layers into the C3D baseline, the network achieves adaptive emphasis on fine-grained spatiotemporal features while accelerating model convergence. In addition, a dedicated Long-Term Spatiotemporal Feature Enhancement Module (LTSFEM) is introduced to effectively address long-sequence dependency modeling, thereby strengthening temporal–causal relationships among actions.
- A lightweight architecture oriented toward edge deployment is designed. To tackle the parameter redundancy and high latency inherent in 3D CNNs, a Channel-Wise Spatiotemporal Block (CWSTB) is proposed for structural reconfiguration, significantly reducing computational overhead while ensuring millisecond-level real-time responsiveness in on-site assembly environments.
- A dedicated dataset and a robust training framework are constructed. The first long-term action dataset for positioning and clamping in aircraft assembly is established, and a combination of the AdamW optimizer and Mixup augmentation is employed to mitigate small-sample overfitting. Comprehensive experiments demonstrate that the proposed method achieves state-of-the-art recognition accuracy and strong generalization robustness in complex industrial scenarios.

The remainder of this paper is organized as follows. Section 2 reviews the related work on action recognition. Section 3 presents the proposed long-term, strongly correlated action recognition network architecture in detail. Section 4 describes the model training strategies and optimization methods. Section 5 reports the experimental setup, result analysis, and ablation studies. Finally, Section 6 concludes the paper and outlines future research directions.

## 2. Related Work

### 2.1. Action Recognition Methods Based on Long Short-Term Memory Networks

To address the limitations of two-stream networks and early 3D CNNs in modeling long-term temporal dependencies, methods based on Long Short-Term Memory (LSTM) networks [2] have emerged as a key technique for capturing long-range contextual dependencies [3,4], owing to their distinctive gating mechanisms. A representative paradigm of such approaches is the “2D CNN + LSTM” framework, in which CNNs are employed to extract high-level frame-wise features, followed by LSTMs to aggregate temporal dynamics. Donahue [5] proposed the Long-term Recurrent Convolutional Networks (LRCN), establishing a cascaded architecture that integrates spatial feature extraction with temporal dynamic modeling. Wu [6] introduced a dual-stream CNN coupled with a regularized feature fusion network, enabling complementary integration of static spatial cues as well as short-term and long-term temporal information. Ullah [7] further enhanced bidirectional temporal modeling of complex dynamic features by employing a deep bidirectional LSTM (DB-LSTM) architecture. To achieve multi-scale spatiotemporal representation, Li [8] developed a multi-stream framework that incorporates LSTMs at the frame, motion, and segment levels to capture hierarchical temporal dynamics. To alleviate the loss of spatial structural information inherent in conventional LSTMs, Li [9] proposed VideoLSTM, which embeds convolutional operations within LSTM units to preserve spatial correlations. Combined with a motion attention mechanism, this approach enables precise focusing on action-sensitive spatiotemporal regions.

### 2.2. Action Recognition Methods Based on 3D Convolutional Neural Networks

Although LSTMs demonstrate strong capability in temporal modeling, their decoupled treatment of spatial and temporal information is not well aligned with the intrinsic spatiotemporal unity of video data. Consequently, an end-to-end modeling paradigm based on three-dimensional convolutional neural networks (3D CNNs) has emerged. Ji et al. [10] were among the first to stack consecutive video frames into spatiotemporal volumes and apply 3D convolutional kernels to jointly extract spatial and temporal features. Subsequently, the C3D network proposed by Tran [11], featuring a homogeneous 3 × 3 × 3 convolutional architecture, demonstrated strong generalization capability and established 3D CNNs as a mainstream approach for video feature extraction. To address the challenges of large parameter size and training difficulty in 3D CNNs, Carreira and Zisserman [1] proposed the two-stream Inflated 3D ConvNet (I3D), which creatively initializes 3D networks using ImageNet-pretrained 2D weights, effectively alleviating the optimization difficulties of deep 3D models. Subsequent studies have primarily focused on improving computational efficiency and architectural optimization. In terms of convolutional factorization, P3D [12], R(2 + 1)D [13], and S3D [14] decouple 3D convolutions into separate spatial and temporal convolutions, significantly reducing parameter complexity while improving performance. Regarding architectural innovations, Res3D [15] incorporates residual connections, and X3D [16] demonstrates the potential of efficient multi-dimensional network scaling. Moreover, to capture higher-order spatiotemporal evolution, the STC module [17] and video-level four-dimensional convolution (V4D) [18] achieve notable advances in feature interaction modeling and explicit long-term temporal modeling, respectively.

### 2.3. Action Recognition Methods Based on Transformers

In recent years, Transformer-based action recognition methods [19] have gained increasing attention due to their superior recognition accuracy. In the exploration of spatiotemporal attention mechanisms, Bertasius [20] proposed TimeSformer based on ViT [21], which employs decoupled spatial and temporal attention to achieve efficient convolution-free video classification. Similarly, the Video Transformer Network (VTN) proposed by Neimark [22] and the Action Transformer introduced by Girdhar [23] abandon conventional 3D CNNs and leverage Transformers to directly capture long-term temporal information and action context across entire video sequences. To overcome the limitation of standard ViT in lacking multi-scale representations, Fan [24] proposed Multiscale Vision Transformers (MViT), which introduce multi-stage hierarchical structures and pooled attention mechanisms to efficiently model multi-granularity visual features without relying on large-scale pretraining. The Swin Transformer proposed by Liu [25] constructs hierarchical feature representations with linear computational complexity through a shifted window mechanism. Subsequently, Swin Transformer V2 [26] further optimized the architecture by enhancing model scalability and adaptability to cross-window resolution variations, achieving state-of-the-art performance on multiple visual benchmark tasks.

### 2.4. Action Recognition in Industrial Scenarios

In the context of intelligent manufacturing, action recognition has become a key enabling technology for monitoring the execution of standard operating procedures and preventing potential assembly quality defects. Although action recognition has been extensively studied in general-purpose scenarios, customized research targeting complex industrial environments, characterized by severe occlusions and fine-grained actions, remains relatively limited [27]. Existing studies in industrial action recognition can be broadly categorized into three groups. The first category focuses on fundamental visual recognition approaches. For instance, Wang [28] constructed an assembly action dataset and verified the effectiveness of 3D convolutional neural networks (3D CNNs) for recognizing industrial assembly actions. The second category incorporates auxiliary information such as tools and human poses. Chen [29] employed tool detection to assist action counting, while Zhang [30] combined CNNs with variable-length Markov models (VMMs) to address prediction challenges in human–machine collaborative scenarios. The third category introduces multimodal data and attention mechanisms to enhance robustness and discrimination capability. Zhao [31] fused electromyography (EMG) signals with channel attention mechanisms to detect non-standard operations. Chen [32] leveraged Longformer to decouple spatiotemporal features and reduce computational complexity, while Gan [33] incorporated skeletal keypoints and attention mechanisms into PoseConv3D, significantly improving the recognition performance for hazardous actions. Giveki [34] proposed a Kernelized Cross-Correlation Convolutional Gated Recurrent Unit (KC^3^GRU) for engine cylinder block assembly action recognition. The method was designed to address the challenge of capturing fine-grained features in industrial action sequences while eliminating the dependence on computationally expensive optical flow estimation.

## 3. Construction of the Positioning and Clamping Action Recognition Model

### 3.1. Problem Formulation and Action Definition

To clarify the boundary between standard short-clip action classification and our proposed task, we formulate a strict mathematical definition for “Long-term Strongly Correlated Action” in the context of aircraft assembly. Let the video stream $V=[I_{1},I_{2},\ldots ,I_{N}]$ represent the continuous image sequence captured in real time from the assembly line, where N is the total number of frames. We parse *v* into a logically coherent action sequence $Act=[a_{1},a_{2},\ldots ,a_{k}]$. Each action instance is defined as a triplet $a_{k}=(c_{k},t_{s,k},t_{e,k})$, where $c_{k}\in C_{long}$ denotes the long-term behavior label, and $t_{s,k}$ and $t_{e,k}$ represent the start and end timestamps of this logical operation, respectively.

An action instance $a_{k}$ is defined as a “long-term strongly correlated action” if and only if it satisfies the following dual constraints:

- Temporal length constraint:

The execution process spans an extended temporal dimension, typically covering a complete functional sub-unit (e.g., positioning or clamping). The duration $\tau_{kL}$ is bounded by a lower threshold $\delta_{\mathrm{L-down}}$ and an upper threshold $\delta_{\mathrm{L-top}}$:

(1) $$\tau_{kL}=t_{e,k}-t_{s,k},\tau_{k}\in [\delta_{\mathrm{L-down}},\delta_{\mathrm{L-top}}]$$

Based on industrial engineering statistics from aircraft assembly sites, we set $\delta_{\mathrm{L-down}}$ = 3.5 s and $\delta_{\mathrm{L-top}}$ = 12 s. Compared to transient actions, this extended timeframe inherently contains multi-stage human–tool–part interactions. This constraint establishes a physical discrimination boundary in the temporal dimension, ensuring the effective extraction of long-range behavioral features.

- Semantic logical strong correlation constraint:

The core characteristic of strongly correlated behavior lies in the irreversibility and logical dependency of the action sequence. The probability of the current action $a_{k}$ occurring is highly dependent on the completion status of the preceding action $a_{k-1}$. This logical dependency is formalized as a conditional probability:

(2) $$P(a_{k}|a_{k-1},a_{k-2},\ldots ,a_{1})\gg P(a_{k})$$

Under the constraints of aircraft assembly procedures, if the preceding “positioning” action is not completed, the subsequent “clamping” action will be strictly identified as a procedural anomaly. This constraint mandates that a strongly correlated action must exist within a specific sequential chain, rather than acting as an isolated, discrete event.

**Figure 2:**
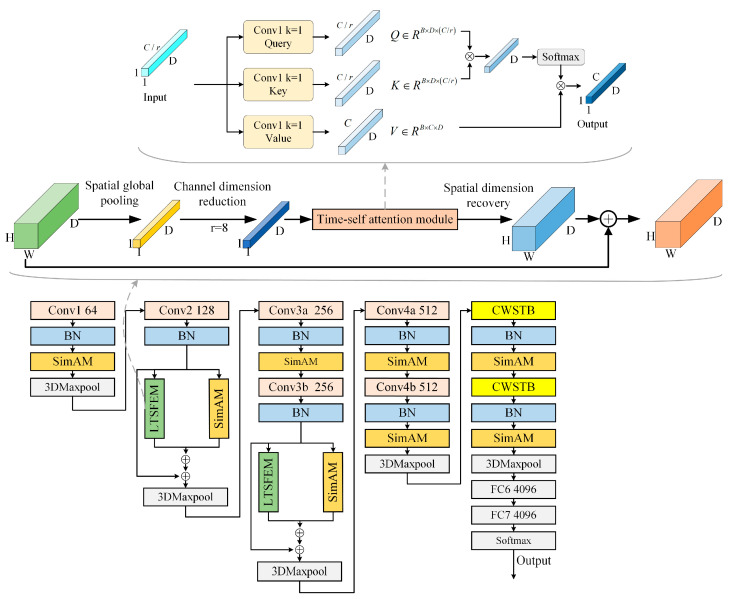
LTSA-Net Architecture Diagram.

The traditional ‘2D CNN + LSTM’ paradigm suffers from spatiotemporal decoupling, thereby limiting its ability to capture subtle motion dynamics. Although Transformer-based architectures exhibit robust global modeling capabilities, their high computational overhead and heavy reliance on massive datasets render them unsuitable for aircraft assembly scenarios characterized by data scarcity and stringent real-time constraints. In contrast, 3D CNNs employ a joint spatiotemporal modeling mechanism that enables the simultaneous extraction of appearance and motion features, making them inherently well-suited to the spatiotemporally unified nature of positioning and clamping actions. Accordingly, this study adopts C3D as the baseline network and proposes LTSA-Net. First, the SimAM module is extended to the 3D domain and integrated with batch normalization (BN) layers, enabling parameter-free adaptive enhancement of spatiotemporal features while accelerating model convergence. More importantly, to address the limited receptive field of convolutional operations, a self-attention-based Long-Term Spatiotemporal Feature Enhancement Module (LTSFEM) is designed. By establishing semantic correlations between temporally distant frames, the proposed module accurately reconstructs the complete operational action logic, as illustrated in Figure 2.

### 3.2. Spatiotemporal Feature Focusing Optimization

In aircraft assembly positioning and clamping scenarios, the accuracy of action recognition critically depends on the effective perception and capture of key spatiotemporal features. However, the original C3D network is constrained by uniform pooling and fixed convolutional kernels, which makes it difficult to dynamically discriminate semantically informative action regions from redundant background noise. To address this limitation, this study introduces the Simple Parameter-free Attention Module (SimAM) [35]. Moreover, SimAM is innovatively extended from the conventional 2D image domain to the 3D spatiotemporal domain, enabling adaptive enhancement of video features. Unlike the original SimAM, which operates solely on spatial dimensions, the proposed 3D SimAM module jointly processes spatiotemporal feature maps $X\in R^{C\times L\times H\times W}$, assigning an attention weight to each neuron within the spatiotemporal volume. The core idea lies in formulating a minimum energy function (Equation (3)), where $\hat{\mu }$ and ${\hat{\sigma }}^{2}$ denote the mean and variance of all neurons within the current channel, respectively; *M* represents the total number of neurons across the spatiotemporal dimensions, and the energy value $e_{t}^{*}$ is negatively correlated with the importance of a neuron. A lower energy value indicates a larger deviation from surrounding background neurons, and thus a higher level of saliency. Finally, the enhancement of the feature map is achieved through Equation (4).

(3) $$e_{t}^{*}=\frac{4\left({\hat{\sigma }}^{2}+\lambda \right)}{{\left(t-\hat{\mu }\right)}^{2}+2{\hat{\sigma }}^{2}+2\lambda }$$

(4) $$\tilde{X}=X⊙sigmoid\left(\frac{1}{E}\right)$$

This energy-driven attention mechanism adaptively reweights feature maps, and the parameter-free nature of SimAM endows it with strong robustness, effectively mitigating the risk of overfitting caused by limited positioning and clamping samples in industrial scenarios. As a result, it enhances the model’s generalization capability while enabling intelligent focusing on critical spatiotemporal cues.

### 3.3. Batch Normalization Model

Positioning and clamping assembly actions are typically conducted in complex industrial environments, where the captured video data inevitably suffers from illumination variations, background interference, and appearance discrepancies caused by camera viewpoints and operator habits. These factors lead to significant shifts in the input data distribution during training, forcing deep networks to continuously adapt to changing distributions, which severely constrains the choice of learning rates and increases the risk of gradient vanishing and overfitting. To address this issue, batch normalization (BN) [36] is adopted in this work. Specifically, BN layers are inserted after each convolutional layer and before the ReLU activation function in the C3D network.

The BN module normalizes the data within each training batch, forcing the input distribution of each layer to have zero mean and unit variance, while introducing learnable parameters to rescale and shift the normalized features, thereby effectively alleviating the internal covariate shift problem. Given an input batch $\eta =\left\{x_{1},x_{2},\ldots ,x_{m}\right\}$, the mean and variance of each feature channel are first computed using Equation (5) and Equation (6), respectively. The input is then normalized according to Equation (7). Finally, Equation (8) applies the learnable parameters γ (scaling factor) and β (shift factor) to adjust the feature distribution, thereby preserving the representational capacity of the network and preventing standardization from diminishing nonlinear characteristics.

(5) $$\mu =\frac{1}{m}\sum_{i=1}^{m}x_{i}$$

(6) $$\sigma^{2}=\frac{1}{m}\sum_{i=1}^{m}{\left(x_{i}-\mu \right)}^{2}$$

(7) $${\hat{x}}_{i}=\frac{x_{i}-\mu }{\sqrt{\sigma^{2}+\varepsilon }}$$

(8) $$y_{i}=\gamma {\hat{x}}_{i}+\beta$$

Through the above mechanisms, the BN module not only stabilizes the input distributions across layers but also effectively smooths the optimization landscape, thereby allowing the use of larger learning rates to accelerate convergence; meanwhile, the batch-wise statistical noise introduced during normalization acts as an implicit regularizer, significantly enhancing the model’s generalization robustness on unseen industrial video data.

### 3.4. Long-Term Spatiotemporal Feature Enhancement Module

In aircraft assembly positioning and clamping processes, key actions often span long temporal intervals, making long-range dependency modeling particularly challenging. Traditional 3D CNNs, constrained by local receptive fields, struggle to effectively capture the complete temporal logic of such operations. To address this issue, a lightweight Long-Term Spatiotemporal Feature Enhancement Module (LTSFEM) is proposed to accurately capture global temporal dependencies under low computational cost.

First, to avoid the prohibitive computational overhead of self-attention on full-resolution feature maps, LTSFEM applies adaptive spatial global pooling to compress the input feature $X\in R^{B\times C\times D\times H\times W}$ along spatial dimensions into $X\in R^{B\times C\times D\times 1\times 1}$, thereby reducing the computational complexity from O(D*(H*W)2) to O(D2), making the cost dependent only on the temporal depth D. Subsequently, a channel reduction mechanism is introduced using a 1 × 1 × 1 convolution to reduce the number of channels from C to C/r. This design not only substantially reduces the parameter count of subsequent linear transformations but also acts as an implicit regularizer by compressing feature representations, effectively alleviating overfitting under small-sample conditions.

Meanwhile, within the compressed feature space, a self-attention mechanism is introduced, where three independent linear transformations are applied to generate the Query, Key, and Value vectors ($Q,K,V\in R^{B\times \left(C/r\right)\times D}$), projecting the features into different representation subspaces. Subsequently, a dot-product operation is employed to compute the inter-frame correlation matrix, and a scaling factor $\sqrt{\left(C/r\right)}$ is introduced to prevent gradient vanishing, as formulated in Equation (9), yielding the attention map $\mathrm{Attention}\in R^{D\times D}$, which quantitatively characterizes the semantic dependencies between any two frames in the video sequence.

(9) $$\mathrm{Attention}=\mathrm{Soft}\max\left(\frac{QK^{T}}{\sqrt{\left(C/r\right)}}\right)$$

Finally, the weighted temporal features are computed using Y_temp=V×Attention and are broadcast back from $\text{Y\_temp}\in R^{B\times C\times D}$ to the original spatial resolution $Y\in R^{B\times C\times D\times H\times W}$ through a spatial broadcasting mechanism. This design ensures that the learned global temporal weights are shared and applied to all spatial locations of each frame. Finally, a residual connection is employed to fuse the enhanced features with the original input. This identity mapping mechanism not only enables the module to be plug-and-play, but also guarantees effective gradient propagation in deep networks, significantly improving training stability and convergence speed.

### 3.5. Architectural Lightweight Optimization

Although C3D exhibits strong performance in action recognition, its parameter count grows quadratically with the number of channels in deeper layers, resulting in severe computational redundancy and an increased risk of overfitting. Inspired by the (2 + 1)D convolution decomposition paradigm, this paper proposes a lightweight Channel-Wise Spatiotemporal Block (CWSTB), which aims to achieve aggressive parameter reduction by decoupling spatiotemporal feature extraction from channel-wise correlation modeling.

Let the input feature tensor be denoted as $X\in R^{C\times T\times H\times W}$. To alleviate channel redundancy in deep feature maps, Equation (10) is employed, where a bottleneck structure is first introduced, applying a 1 × 1 2D convolution to project the input features into a lower-dimensional space. Given a compression ratio τ, the output channel dimension is reduced to $C_{red}=[C/\tau ]$, which significantly reduces the subsequent computational cost while also serving as an effective feature selection mechanism.

(10) $$X_{red}=F_{1\times 1}^{2D}(X)\in R^{C_{red}\times T\times H\times W}$$

To efficiently capture the temporal evolution of actions in a low-dimensional feature space, a channel-grouped temporal convolution is further designed. Specifically, the spatial dimensions (H,W) of the input feature map are first flattened, preserving only the temporal and channel-wise information. Subsequently, a one-dimensional convolution is applied along the temporal axis to explicitly model the dynamic variations in actions over time. To achieve extreme model lightweighting and computational efficiency, the number of convolution groups is set equal to the number of input channels $C_{red}$, thereby forming a depthwise separable temporal convolution, as formulated in Equation (11). This design significantly reduces the parameter count and computational overhead while maintaining fine-grained modeling capability of channel-level temporal features, effectively enhancing the model’s suitability for resource-constrained industrial scenarios.

(11) $$X_{temp}^{\left(k\right)}=Conv1D(X_{red}^{\left(k\right)}),\;k\in \{1,\ldots ,C_{red}\}$$

Finally, after modeling the temporal dependencies, a standard 2D convolution ($k_{s}=3$) is applied to $k_{s}\times k_{s}$ to restore local spatial correlations, while projecting the channel dimension back to $C_{out}$, as formulated in Equation (12).

(12) $$Y=\sigma (\mathrm{BN}(F_{k_{s}\times k_{s}}^{2D}(X_{\mathrm{temp}})))$$

To validate the effectiveness of the proposed CWSTB, its parameter complexity is compared with that of a standard 3D convolution. Assuming that both the input and output channel numbers are C, and the spatiotemporal kernel sizes are $k_{t}$ and $k_{s}$, respectively, the parameter count of the standard 3D convolution is given in Equation (13), while that of the CWSTB is formulated in Equation (14).

(13) $$P_{C3D}\approx C^{2}\cdot k_{t}\cdot k_{s}^{2}$$

(14) $$P_{\mathrm{CWSTB}}\approx \frac{C^{2}}{\tau }+\frac{C}{\tau }\cdot k_{t}+\frac{C^{2}}{\tau }\cdot k_{s}^{2}$$

Since τ is typically set to a relatively large value (16 in this study), and the intermediate temporal convolution adopts a depthwise separable strategy, reducing the parameter count from $\frac{C^{2}}{\tau }$ to $\frac{C}{\tau }$, the overall parameter complexity is significantly decreased, resulting in $P_{CWSTB}\ll P_{C3D}$. This design enables the replacement of computationally expensive 3D convolution layers with stacked CWSTBs in the deep Conv5 stage of the network, thereby substantially improving the model’s inference efficiency.

## 4. Datasets and Evaluation Settings

To address the severe data scarcity in aircraft positioning and clamping assembly scenarios, this study constructs a dedicated dataset tailored to aircraft assembly positioning and clamping actions. In conjunction with data augmentation strategies and an efficient training protocol, the proposed framework enables rapid model convergence and enhanced robustness under small-sample conditions, thereby meeting the requirements for real-time monitoring and iterative model deployment.

### 4.1. Optimizer Improvement Strategy

The original C3D model utilizes the Stochastic Gradient Descent (SGD) optimizer, which updates parameters by randomly selecting individual instances or mini-batches. This optimization scheme inevitably introduces gradient noise, causing parameter trajectories to deviate from the global optimum. Moreover, in aircraft positioning and clamping assembly scenarios, disturbances such as abrupt illumination changes and mechanical vibrations further exacerbate gradient bias, requiring SGD to perform a large number of iterations to approach the global optimum. To address these limitations, this study introduces the AdamW optimizer [37], which leverages first-order moment estimation (momentum) and second-order moment estimation (adaptive learning rates) to smooth the optimization trajectory. Compared with Adam, AdamW decouples weight decay from gradient updates: instead of incorporating the weight decay term into the gradient computation, it applies weight decay directly during the parameter update step by independently subtracting the decay term from the current parameter values. The parameter update rule is given in Equation (15).

(15) $$\theta_{t+1}=\theta_{t}-\eta \left(\frac{{\hat{m}}_{t}}{\sqrt{{\hat{v}}_{t}}+\varepsilon }\right)-\eta \lambda \theta_{t}$$

where λ denotes the independent weight decay coefficient, and $\lambda \theta_{t}$ represents the weight decay term. Weight decay, as a classical regularization technique, introduces the L2 norm of the model weights as a penalty term to constrain parameter magnitude, thereby effectively mitigating the risk of overfitting. AdamW decouples the weight decay term from the gradient computation, allowing it to act directly on the parameter update step rather than being implicitly embedded in the gradient. Through this explicit decay mechanism, model complexity can be more effectively controlled, leading to improved generalization performance and enhanced robustness against noise disturbances in aircraft positioning and clamping assembly scenarios.

### 4.2. Dataset Configuration

**Figure 3:**
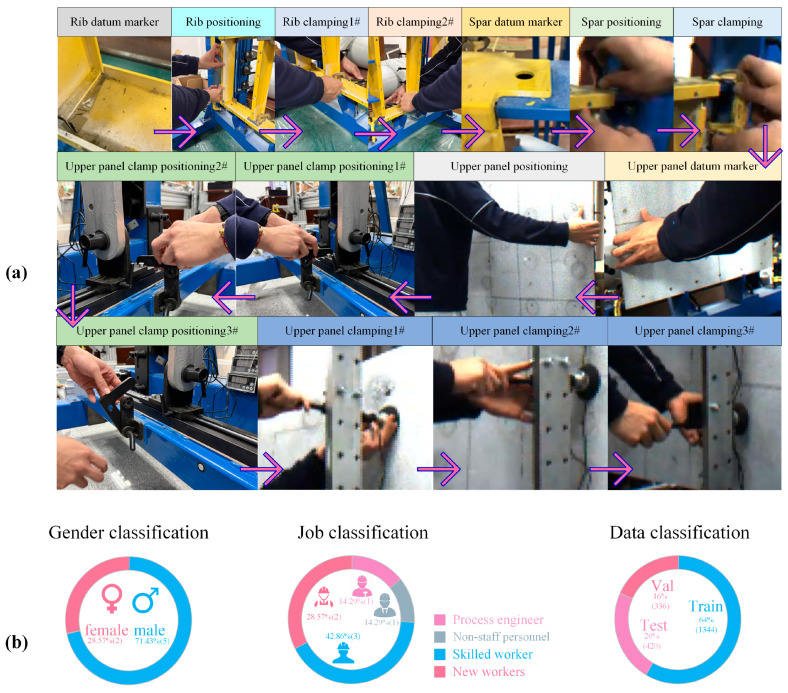
Detailed examples of each class in the LTSA-Dataset, (**a**) represents the specific workflow of the dataset, and (**b**) represents the classification of the dataset’s data.

The LTSA-Dataset is a highly specialized industrial dataset specifically designed for aircraft positioning and clamping assembly tasks. It comprises 2100 video samples collected from seven skilled assembly operators, as illustrated in Figure 3. In accordance with the aircraft positioning and clamping workflow, the dataset was meticulously annotated into 15 core action categories: 1. Rib datum marker; 2. Rib positioning; 3. Rib clamping1#; 4. Rib clamping2#; 5. Spar datum marker; 6. Spar positioning; 7. Spar clamping; 8. Upper panel datum marker; 9. Upper panel positioning; 10. Upper panel clamp positioning1#; 11. Upper panel clamp positioning2#; 12. Upper panel clamp positioning3#; 13. Upper panel clamping1#; 14. Upper panel clamping2#; 15. Upper panel clamping3#. The total duration of the dataset is 142.01 min. Video clip lengths range from 2.21 s to 6.89 s, with an average duration of 4.03 s (as shown in Table 1). The videos were recorded at multiple resolutions, including 720 × 540, 1280 × 1024, and 2448 × 2047, and are stored in MP4 format.

As illustrated in Figure 3, the LTSA-Dataset exhibits the following notable advantages:

1. High domain specificity. The LTSA-Dataset is currently the largest industrial action dataset dedicated to aircraft assembly positioning and clamping tasks. It comprises 2100 video instances collected from seven operators, all acquired under real manufacturing process conditions. This ensures strong ecological validity and enhances the model’s generalization capability in real-world deployment scenarios.
2. Diverse participants. The selection of operators was constrained by job roles and proficiency levels, including two novice operators (28.57%), three skilled operators (42.86%), one process engineer (14.29%), and one external participant (14.29%). In addition, the gender distribution consists of five males (71.43%) and two females (28.57%), further increasing inter-subject variability. Meanwhile, the dataset was divided according to a 16:4:5 ratio for the training, validation, and test sets, respectively. Notably, all data collected from novice operators were exclusively assigned to the test set.
3. Heterogeneous data formats. Videos were captured using cameras with three different resolutions, enabling flexible frame-rate and resolution configurations to accommodate different network architectures and computational constraints in subsequent experiments.

Compared with existing datasets, LTSA-Dataset demonstrates clear advantages in terms of action categories, number of action instances, participant diversity, and video formats. It fills a critical gap in research on aircraft assembly positioning and clamping action recognition, while its controlled yet realistic variability provides a solid foundation for improving model generalization performance. Furthermore, similar long-term strongly associated assembly actions are widely observed in other advanced manufacturing domains, such as automotive assembly and high-speed rail component assembly. Therefore, the proposed framework has strong potential for generalization beyond aircraft assembly and can be readily extended to other assembly scenarios. More broadly, it can be applied to a wide range of industrial monitoring tasks characterized by sequential operations, procedural constraints, and standardized workflows.

### 4.3. Mixup Data Augmentation Strategy

To address the challenges of sample scarcity, class imbalance, and complex backgrounds in aircraft assembly positioning and clamping tasks, this study introduces the Mixup data augmentation strategy [38]. Unlike traditional geometric transformations, Mixup is grounded in the principle of vicinal risk minimization, generating virtual training samples by performing convex combinations of both input samples and their corresponding labels, thereby effectively expanding the support of the training distribution. Specifically, two samples, denoted as $\left(x_{i},y_{i}\right)$ and $\left(x_{j},y_{j}\right)$, are randomly selected from a mini-batch, and a mixed sample $\left(\tilde{x},\tilde{y}\right)$ is generated according to Equations (16) and (17).

(16) $$\lambda ∼Beta\left(\alpha ,\alpha \right)$$

(17) $$\tilde{x}=\lambda x_{i}+\left(1-\lambda \right)x_{j},\;\tilde{y}=\lambda y_{i}+\left(1-\lambda \right)y_{j}$$

**Figure 4:**
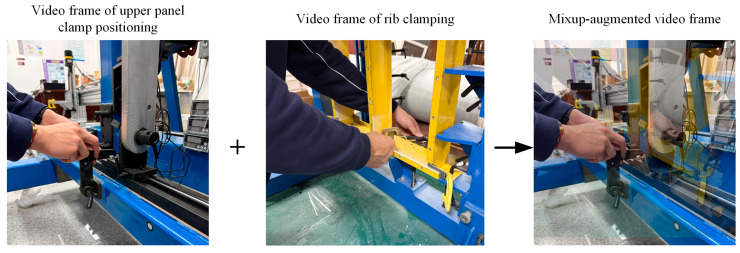
Mixup data augmentation examples.

Here, the mixing coefficient λ∈[0,1] follows the distribution $Beta\left(\alpha ,\alpha \right)$, where the hyperparameter *α* controls the strength of interpolation. Empirical results from both practical application and experimental evaluation indicate that the choice of this parameter has a significant impact on training performance. Specifically, the value of *α* directly affects the degree of mixing between two behavior sequences. Smaller values of *α* generally lead to better performance, whereas excessively large values result in marginal performance gains or even degradation. This is because over-mixing disrupts the inherent spatiotemporal structure of the original behavior sequences, thereby weakening discriminative visual cues. Accordingly, the parameter *α* is set to α=0.2 in this study. The effect of Mixup-based augmentation is illustrated in Figure 4.

The introduction of the Mixup strategy provides a significant regularization effect for the model. By constructing linear transitions between training samples, Mixup smooths the decision boundaries between classes. This not only effectively alleviates overfitting caused by limited training data but also substantially enhances the model’s intrinsic robustness in real aircraft manual assembly environments, enabling it to better withstand severe illumination variations, significant local physical occlusions, and frequent hand–tool interaction disturbances.

## 5. Experiment Verification

To demonstrate the superiority of LTSA-Net in aircraft assembly positioning and clamping behavior recognition, extensive experiments were conducted on the LTSA-Dataset. The performance of LTSA-Net was compared with that of mainstream action recognition networks, followed by ablation studies to evaluate the contribution of each proposed module. Finally, qualitative visualization analyses were performed to further assess and interpret the performance of LTSA-Net.

### 5.1. Evaluation Metrics and Experimental Details

To ensure a comprehensive and objective evaluation of model performance, Top-1 Accuracy is adopted as the primary evaluation metric. This metric directly reflects the model’s ability to correctly classify aircraft assembly video clips into their corresponding behavior categories. Given a dataset containing N samples, Top-1 Accuracy is defined as the proportion of samples for which the predicted class with the highest probability matches the ground-truth label. The calculation is given in Equation (18), where ${\hat{y}}_{i}$ denotes the predicted class of the i-th sample, $y_{i}$ represents the corresponding ground-truth label, and $I\left(\cdot \right)$ is the indicator function.

(18) $$\mathrm{Top-1}\;\mathrm{Accuracy}=\frac{1}{N}\sum_{i=1}^{N}I\left({\hat{y}}_{i}=y_{i}\right)$$

In addition, to verify the feasibility and efficiency of deploying LTSA-Net in real industrial environments, this study introduces floating-point operations (FLOPs) and frames per second (FPS) as evaluation metrics to measure the model’s computational complexity and real-time inference capability, respectively. All experiments were conducted on a unified hardware and software platform, with detailed configurations listed in Table 2.

During training, the network was trained for 100 epochs, with the learning rate set to 0.0001, batch size set to 16, and dropout rate set to 0.5. The cross-entropy loss was used as the objective function. Regarding optimization strategies, when the SGD optimizer was adopted, the momentum was set to 0.9 and the weight decay factor to 5 × 10^−4^. When using the AdamW optimizer, the weight decay factor was also set to 5 × 10^−4^.

### 5.2. Training Strategy Optimization Experiments

To evaluate the independent contributions of training strategies beyond network architecture improvements, the C3D network is adopted as the baseline. Based on this baseline, the effectiveness of the AdamW optimizer and the Mixup data augmentation strategy is investigated for the aircraft assembly positioning and clamping action recognition task.

#### 5.2.1. Optimizer Comparison Experiments

**Figure 5:**
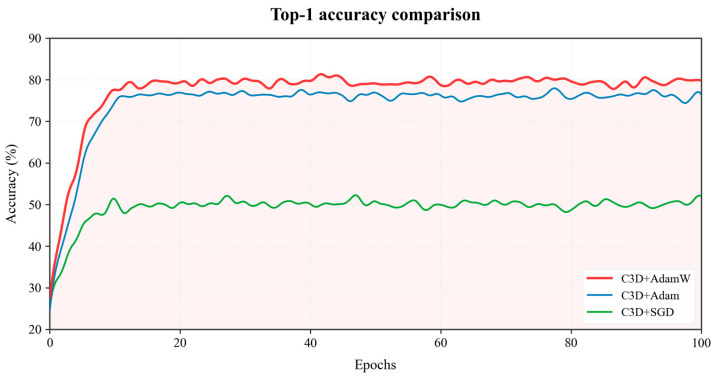
Training accuracy convergence curves for different optimizers.

To ensure fairness, all experiments in this section are conducted based on the standard C3D network, without introducing any of the additional modules proposed in this paper. Table 3 reports the performance of the baseline C3D model using three different optimizers on the LTSA-Dataset, while Figure 5 illustrates the corresponding accuracy convergence curves during training. The experimental results indicate that the AdamW optimizer achieves the best performance, outperforming SGD and Adam by 27.94% and 2.85%, respectively. This performance advantage can be attributed to AdamW’s decoupled weight decay mechanism, which separates weight decay from gradient updates. Such a design effectively alleviates overfitting and significantly enhances the model’s generalization capability in complex aircraft assembly action recognition tasks.

#### 5.2.2. Mixup Data Augmentation Strategy Experiments

**Figure 6:**
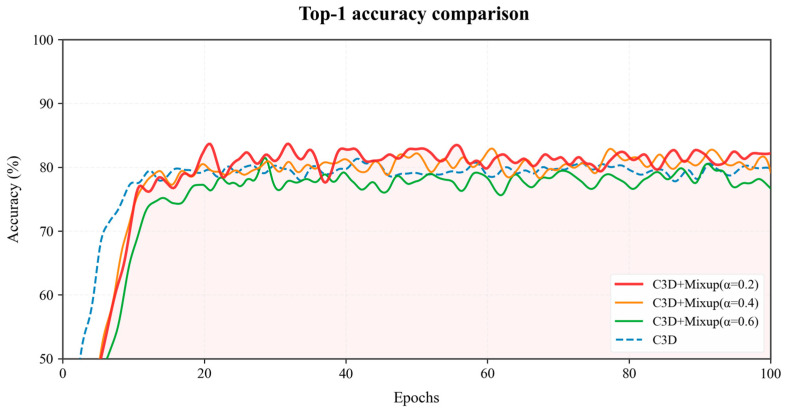
Training accuracy convergence curves for the Mixup data augmentation strategy.

Mixup constructs virtual training samples via linear interpolation, encouraging the model to learn linear decision boundaries between samples. Considering that the Mixup parameter α in image classification tasks is typically set within the range of 0.2–0.4 [39], this study evaluates α values from 0.2 to 0.6 on the LTSA-Dataset using the AdamW optimizer. Table 4 reports the impact of different α values on model performance, while Figure 6 illustrates the corresponding training curves under different Mixup settings. The experimental results demonstrate that introducing the Mixup strategy consistently improves performance across all settings compared with the baseline model, confirming its effectiveness in smoothing decision boundaries and enhancing robustness. Specifically, when α = 0.2, the Top-1 accuracy increases by 2.93%, yielding the most significant performance gain. In contrast, when α = 0.6, the accuracy improvement is limited to 1.67%, indicating diminishing returns under excessive mixing.

### 5.3. Ablation Study of Model Components

To verify the effectiveness of each proposed improvement in this chapter, systematic ablation experiments were conducted. All experiments were performed under identical data splits and experimental settings, with the original C3D network serving as the baseline model. Each module was then incrementally introduced to evaluate its individual contribution to overall performance.

#### 5.3.1. Batch Normalization Module Embedding Experiment

In this subsection, the BN module is embedded into the baseline C3D network to independently evaluate its effectiveness. Specifically, a BN layer is inserted after each 3D convolution layer and before the activation function in the baseline architecture. All experiments are conducted using the AdamW optimizer. The modified network is trained and evaluated on the LTSA-Dataset, and the corresponding accuracy results are reported in Table 5.

**Figure 7:**
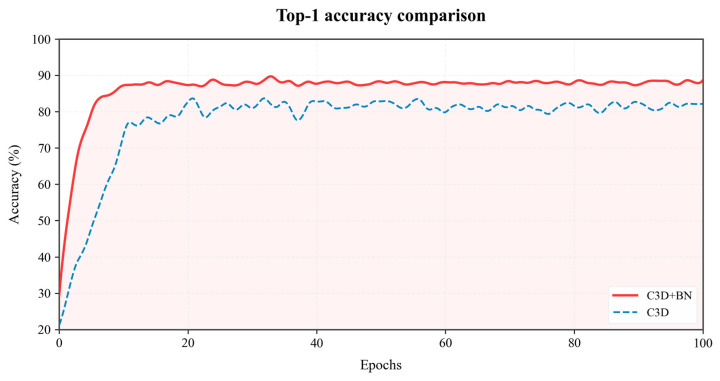
Training accuracy curves for the BN module embedding experiment.

According to [39], embedding the BN modules into the C3D network improves the recognition accuracy to 89.83%, representing an increase of 5.78% over the baseline. This result clearly demonstrates that BN stabilizes the distribution of internal activations, enabling the model to learn more discriminative feature representations and thereby enhancing its classification capability. The comparison of training accuracy curves and training loss curves is illustrated in Figure 7.

By observing the accuracy curves, it can be seen that the baseline network exhibits significant fluctuations, indicating instability during training. In contrast, the network with embedded BN modules shows a much smoother training curve, highlighting the stabilizing effect of the BN module on the training process.

#### 5.3.2. SimAM Attention Module Embedding Experiment

To investigate the optimal embedding strategy of the parameter-free SimAM attention module within a 3D convolutional network, this experiment uses C3D + BN as the baseline and focuses on the effects of embedding position (before or after pooling layers) and embedding quantity on spatiotemporal feature extraction. Three configurations were designed:

1. C3D + SimAM1: 5 SimAM modules embedded after the pooling layers;
2. C3D + SimAM2: 5 SimAM modules embedded before the pooling layers;
3. C3D + SimAM3: 8 SimAM modules embedded before the pooling layers.

The experimental results are shown in Table 6.

**Figure 8:**
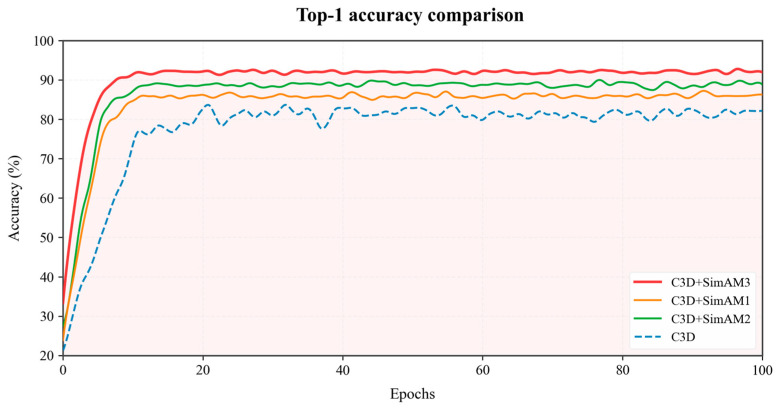
Training accuracy curves for the SimAM attention module embedding experiment.

The accuracy curves of the four network configurations during training are shown in Figure 8. The results indicate that all models incorporating the SimAM module outperform the baseline network, with C3D + SimAM3 achieving the highest accuracy of 93.02%, representing an 8.97% improvement over the original C3D. This demonstrates that the SimAM module can guide the network to focus on critical spatiotemporal features in the video via its energy function, thereby enhancing recognition accuracy.

Further analysis shows that embedding SimAM before the pooling layer (SimAM2) significantly outperforms embedding after the pooling layer (SimAM1), with an accuracy gain of 2.86%. This is because pooling operations can result in the loss of some spatial details. Introducing the attention mechanism before pooling allows SimAM to compute its energy function based on the original feature maps, strengthening key spatiotemporal features before downsampling and preserving crucial information. Comparing SimAM2 and SimAM3, increasing the number of embedded modules from 5 to 8 yields an additional performance improvement (+2.26%). This indicates that applying attention consecutively across more convolutional layers, especially from shallow to deep layers, can construct a multi-scale feature refinement mechanism.

#### 5.3.3. Long-Term Sequence Feature Enhancement Module Embedding Experiment

**Figure 9:**
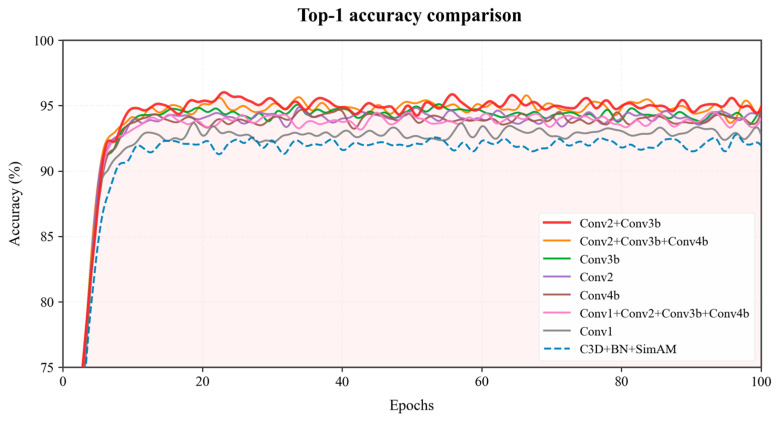
Training accuracy curves for LTSFEM embedding experiment.

This section employs C3D + BN + SimAM (8 blocks) as the baseline network to investigate the optimal collaboration strategy of the dual-path attention mechanism formed by LTSFEM and SimAM. Considering that assembly actions exhibit multi-scale temporal dependencies, this experiment focuses on evaluating the embedding effects of LTSFEM at different convolutional layers, aiming to balance temporal modeling capability with computational complexity. The experiment considers seven embedding configurations covering Conv1 to Conv4b: (1) Conv1: embedding after the Conv1 layer; (2) Conv2: embedding after the Conv2 layer; (3) Conv3b: embedding after the Conv3b layer; (4) Conv4b: embedding after the Conv4b layer; (5) Conv2 + Conv3b: embedding after both Conv2 and Conv3b layers; (6) Conv2 + Conv3b + Conv4b: embedding after Conv2, Conv3b, and Conv4b layers; (7) Conv1 + Conv2 + Conv3b + Conv4b: embedding after Conv1, Conv2, Conv3b, and Conv4b layers. As shown in Table 7, the performance improvements of the baseline network after embedding LTSFEM at different convolutional layers are presented, and Figure 9 compares the accuracy curves of the baseline network with LTSFEM embedded at multiple convolutional layers.

As shown in Table 7, embedding in the shallow layer (Conv1) only yields a 0.77% improvement. Since shallow features are primarily composed of low-level visual primitives and noise, lacking explicit semantic information, LTSFEM struggles to extract meaningful long-term sequential patterns. Conversely, embedding LTSFEM in all convolutional layers results in only a 1.77% improvement, as excessive LTSFEM introduces redundant temporal attention, substantially increasing computational cost and interfering with feature learning. The optimal performance is achieved when LTSFEM is embedded only after the Conv2 and Conv3b layers, reaching an accuracy of 96.22%, which represents a 3.20% improvement over the baseline. Analysis indicates that the Conv2 and Conv3b layers contain discriminative mid- to high-level semantic information while retaining sufficient temporal resolution, making them suitable for fine-grained long-term sequential modeling. SimAM is responsible for precisely localizing key regions in the spatial dimension, while LTSFEM in Conv2/3b captures the evolution of these key regions over extended temporal domains. The two components complement each other, achieving deep decoupling and enhancement of spatiotemporal features.

#### 5.3.4. Lightweight Module Embedding Experiment

**Figure 10:**
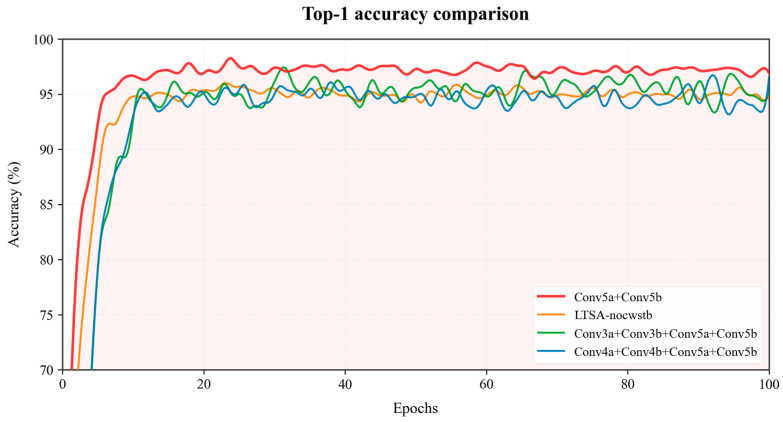
Training accuracy curves for the lightweight module.

In this experiment, the baseline network is a C3D network with BN modules embedded, SimAM parameter-free attention modules (eight modules in total) inserted after convolutional layers and before pooling layers, and LTSFEM embedded in the Conv2 and Conv3b layers (denoted as LTSA-nocwstb). Building on previous experiments, this study aims to explore the optimal embedding strategy for the lightweight CWSTB module. The experiment systematically evaluates how the embedding position and number of CWSTB modules affect network performance, with the goal of reducing model parameters. Three configurations are considered: (1) Conv5a + Conv5b: replacing Conv5a and Conv5b; (2) Conv3a + Conv3b + Conv5a + Conv5b: replacing Conv3a, Conv3b, Conv5a, and Conv5b; (3) Conv4a + Conv4b + Conv5a + Conv5b: replacing Conv4a, Conv4b, Conv5a, and Conv5b. Table 8 presents the experimental results for each configuration, and Figure 10 shows the corresponding training curves.

Detailed analysis indicates that replacing only the last two convolutional layers achieves the highest accuracy of 98.24%, representing a 2.02% improvement over the non-lightweight version. This suggests that deep convolutional networks often contain substantial parameter redundancy. The CWSTB module not only significantly reduces parameter count but also enhances feature interactions, thereby improving the model’s generalization capability. Additional replacement of Conv3 or Conv4 layers results in performance degradation. This is because the middle layers are responsible for constructing core semantic features; excessive lightweight operations restrict the model’s feature extraction capacity, causing key information to be lost during channel fusion. Applying CWSTB at Conv5a and Conv5b achieves the optimal balance between parameter efficiency and recognition accuracy, demonstrating the effectiveness of this lightweight design.

#### 5.3.5. Performance Comparison of Individual Modules

To quantitatively evaluate the individual and cumulative contributions of the introduced components to the improved model, a series of progressive ablation experiments were conducted using the original C3D network as the baseline. All experiments were performed on the same benchmark dataset under identical experimental settings. The Top-1 accuracy achieved by different module configurations, along with their corresponding performance gains, is presented in Table 9.

As shown by the quantitative results in Table 4, the proposed modules work synergistically and consistently contribute to performance improvements over the baseline network, ultimately achieving the highest accuracy of 98.24% on the benchmark dataset. First, after introducing the BN layer, the Top-1 accuracy increased from 84.05% to 89.93%, representing a substantial improvement of 5.88%. This result indicates that BN effectively alleviates the problem of internal covariate shift in deep 3D convolutional networks during training. By stabilizing gradient propagation, BN significantly enhances the robustness of low-level feature representations. Subsequently, incorporating the parameter-free SimAM attention mechanism further improved the accuracy by 3.09%. Given the complex backgrounds, illumination variations, and occlusion disturbances commonly encountered in aircraft assembly workshops, SimAM enables the network to adaptively focus on informative spatiotemporal regions without introducing additional computational overhead, thereby effectively suppressing background noise in industrial environments. Notably, the LTSFEM yielded a further performance gain of 3.20%, increasing the accuracy to 96.22%. This substantial improvement strongly validates the effectiveness of LTSFEM in capturing long-range temporal dependencies and contextual information. Such capability is particularly critical for accurately distinguishing continuous assembly operations characterized by strong temporal correlations and long-duration procedural dependencies. Finally, by integrating the CWSTB, the model achieved a peak accuracy of 98.24% on the benchmark dataset, corresponding to an additional improvement of 2.02%. This result demonstrates the effectiveness of CWSTB in refining cross-dimensional spatiotemporal interactions and reducing feature redundancy, thereby further enhancing the overall discriminative capability of the proposed framework.

### 5.4. Comparison with Mainstream Models

To comprehensively evaluate the performance of LTSA-Net in aircraft assembly scenarios, this section benchmarks it against two categories of mainstream video action recognition methods: (1) Convolutional Neural Networks (CNNs), including the C3D baseline model, the two-stream I3D architecture, and the SlowFast model; and (2) Vision Transformers, including Swin Transformer V2 [26] and TimeSformer [20]. To ensure fair comparison, all experiments were conducted on the LTSA-Dataset under a consistent hardware environment. In addition to recognition accuracy, this section introduces three metrics: number of parameters (Params), floating-point operations (FLOPs), and frames per second (FPS), to quantitatively assess the efficiency and feasibility of deploying the models in industrial environments. Among them, the FLOPs were calculated based on a standard input size of 3 × 16 × 112 × 112 (C × D × H × W), corresponding to an input resolution of 112 × 112 and a clip length of 16 frames. The detailed comparison results for all models are presented in Table 10.

Experimental results indicate that although Swin Transformer V2 achieves a high accuracy of 94.60% through its self-attention mechanism, LTSA-Net attains 98.24%, outperforming it by 3.64%. This strongly demonstrates that the proposed LTSFEM module is superior to general Transformer architectures in capturing fine-grained industrial action features. In terms of efficiency, LTSA-Net reduces the number of parameters by 45% compared to TimeSformer and achieves three times faster inference. Moreover, its high frame rate of 98 FPS approaches that of pure convolutional networks (SlowFast, 105 FPS). Meanwhile, all data collected from novice operators were exclusively used as the test set during model training. The rationale behind this design is that, during assembly operations, the behavioral characteristics of inexperienced operators often closely resemble fatigue-induced errors observed during prolonged work periods. These behaviors typically manifest as occasional operational pauses, unstable motion trajectories, and abnormal extensions of standard action cycles. Benefiting from the LTSFEM module’s capability to capture global temporal dependencies and the 3D SimAM mechanism’s ability to suppress background noise, LTSA-Net maintained an exceptionally high recognition accuracy (98.24%) when processing behavioral sequences characterized by rhythm distortions and operator heterogeneity. These results demonstrate that the proposed framework can effectively cope with operator variability and fatigue-induced behavioral deviations through its inherent robustness, without requiring additional complex modules, thereby fully satisfying the robustness requirements of quality monitoring in practical assembly environments. In summary, LTSA-Net successfully overcomes the limitations of CNNs in long-term sequential modeling, achieving state-of-the-art accuracy while maintaining high throughput, making it the most effective solution for aircraft assembly positioning and clamping scenarios.

### 5.5. Visualization Analysis

**Figure 11:**
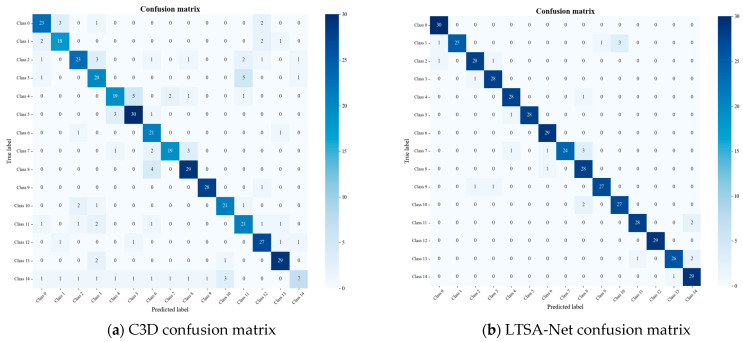
Confusion matrix.

To clearly demonstrate the performance advantages of LTSA-Net in recognizing long-term strongly associated behaviors, this section presents a comparative analysis of the confusion matrices obtained by the baseline model (C3D) and the proposed model (LTSA-Net), as shown in Figure 11. In addition, the classification performance of LTSA-Net on the test set is summarized in Table 11.

**Figure 12:**
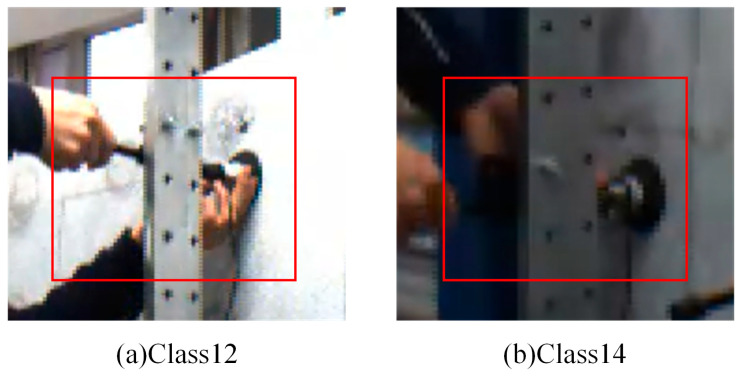
Comparison of Behavioral Scenarios.

The comparison presented in Figure 11 provides a direct visualization of the performance improvement achieved by the proposed architecture. The baseline model exhibits noticeable decision-boundary ambiguity and scattered misclassifications when recognizing long-duration actions. In contrast, benefiting from the noise-suppression capability of the SimAM module and the global temporal modeling ability of LTSFEM, the confusion matrix of LTSA-Net demonstrates a significantly stronger concentration along the diagonal, indicating a substantial improvement in classification consistency. Crucially, as this testing set consists exclusively of unseen novice operators, these metrics serve as a high-fidelity, process-level stress test for both cross-subject generalization and skill-level robustness. According to the statistical results shown in Table 11, LTSA-Net achieves excellent classification balance across all 15 action categories. Notably, Class 12 (Upper panel clamping 1#) achieves perfect recognition performance, with all evaluation metrics reaching 100%. In addition, Classes 0, 5, 6, and 7 achieve 100% precision or recall. These results clearly demonstrate the enhanced capability of the proposed model to discriminate fine-grained actions in complex industrial environments. However, although Class 14 shares highly similar geometric motion trajectories with Classes 12 and 13, its recognition performance remains slightly lower than that of the other two categories, with an F1-score of 92.06%. To further investigate the underlying cause of this performance discrepancy, a comparative analysis of the real operational viewpoints was conducted, as shown in Figure 12.

A comparison of the key interaction regions highlighted by the red boxes in Figure 12a,b reveals that, although the two actions exhibit highly similar motion trajectories, Class 12 is performed in a well-illuminated area. The operator’s hand posture, the contour of the pneumatic tool, and the interaction contact point between the tool and the panel all exhibit strong visual contrast, which facilitates the lossless extraction of discriminative spatiotemporal features by the model. In contrast, the operational location of Class 14 is situated in the deepest section of the wing-box assembly area. The narrow cavity severely obstructs external light sources, resulting in an extremely dark scene within the highlighted region. The combined effect of structural spatial occlusion and severe illumination attenuation leads to a substantial reduction in image signal-to-noise ratio, causing the physical loss of fine-grained edge and texture features that are critical for behavior recognition. Consequently, a limited degree of feature ambiguity is introduced. Nevertheless, even under these harsh physical constraints, where visual information is significantly degraded, the proposed model maintains a high level of classification reliability. Benefiting from the noise-suppression capability of the SimAM module and the global temporal recovery ability of LTSFEM, LTSA-Net does not produce any false-positive predictions for Class 14. The observed performance degradation is primarily attributable to a small number of false negatives caused by incomplete feature information. These results further validate the robustness and effectiveness of the proposed framework for recognizing long-term strongly associated assembly behaviors under challenging industrial conditions. To further mitigate the impact of spatial occlusions, future work will investigate the integration of multi-view visual fusion mechanisms and first-person wearable vision systems (e.g., AR-glass cameras) as auxiliary input sources. By leveraging complementary information from multiple viewpoints, the inherent blind spots of monocular vision systems can be effectively eliminated, thereby meeting the stringent industrial requirements of aircraft assembly action recognition.

### 5.6. Real-World Scenario Validation

**Figure 13:**
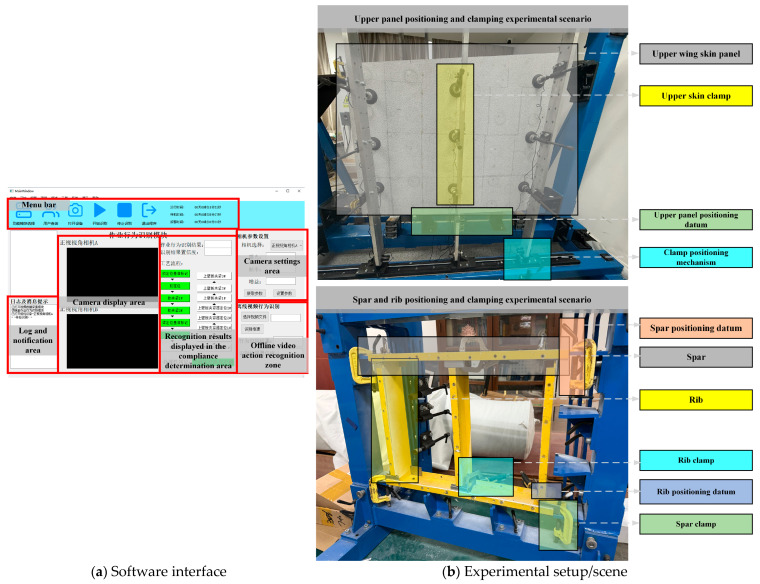
Real-world scenario validation figure.

To validate the system’s performance in real aircraft assembly scenarios, a real-time inference testing environment based on industrial camera video streams was established. The experiment deployed two front-facing cameras (Camera A/B) in the spar/rib and upper panel positioning and clamping areas to capture operational footage. The system processes the video streams in real time and outputs the Top-1 predicted action class (hardware configuration and software interface are shown in Table 12 and Figure 13, respectively). The test dataset consists of 50 complete assembly videos and several isolated action clips collected by three operators at different times, covering all 15 predefined action classes. The aim is to comprehensively assess the system’s recognition accuracy and real-time responsiveness. The software configuration used in the experiments is summarized in Table 2. To faithfully simulate real-world edge deployment conditions, where video frames captured by industrial cameras are processed sequentially and require immediate real-time decision-making, the batch size was set to **1** throughout the experiments.

The system achieves an overall recognition accuracy of 98.82% in the simulated assembly scenario (detailed results are shown in Table 13). The recognition rates for positioning and clamping actions related to ribs, beams, and upper panels all exceed 98.5%, demonstrating the algorithm’s effectiveness and reliability.

Error analysis indicates that the recognition accuracy for “Rib Clamping #2” and “Upper Panel Clamp Positioning #3” is relatively low. The primary reasons are: (1) Scene occlusion: the limited workspace obstructs part of the field of view; (2) Feature confusion: related actions exhibit minimal visual differences from certain perspectives or are highly similar to actions in other locations, leading to ambiguous features. Future work will incorporate part assembly as semantic guidance to collaboratively recognize worker actions, aiming to further reduce misclassifications. Real-time stream testing (see Table 14) shows that the system achieves an average processing frame rate of 28 FPS, matching the frame rates of mainstream industrial cameras and ensuring low-latency video stream processing. The average recognition time of 42 ms and peak time of 54 ms further validate the algorithm’s efficiency and robustness under prolonged operation.

In summary, the system exhibits excellent real-time responsiveness, fully meeting the stringent requirements of intelligent monitoring in aircraft assembly environments.

## 6. Conclusions

As critical phases in the aircraft assembly process, positioning and clamping demand high operator proficiency and strict procedural compliance. Omissions, non-standard operations, or fatigue-induced errors can easily compromise the integrity of subsequent assembly processes. To address these vulnerabilities, this study developed an intelligent vision-based verification framework specifically tailored for manual positioning and clamping operations. The primary research conclusions are summarized as follows:

- The positioning and clamping process was theoretically defined as a “long-term strongly correlated action.” By elucidating the spatiotemporal evolution and contextual dependencies of fine-grained sub-actions, this study establishes a solid foundation for high-precision action modeling.
- A high-efficiency model, LTSA-Net, was proposed. It optimizes the C3D backbone by integrating SimAM and BN modules to enhance feature focus and stability, employing LTSFEM to capture global temporal dependencies, and utilizing CWSTB for lightweight parameter compression. This design effectively balances recognition accuracy with inference efficiency.
- Leveraging the custom LTSA-Dataset and a robust training framework (AdamW/Mixup), LTSA-Net achieved an accuracy of 98.82% with an inference latency of 42 ms. These metrics demonstrate that the model successfully reconciles the trade-off between high precision and real-time demands, offering a viable paradigm for intelligent industrial monitoring.

Although the proposed LTSA-Net achieves significant performance in aircraft assembly positioning and clamping scenarios, there remains room for further optimization in complex industrial manufacturing environments. Future research will focus on the following three aspects:

- Model transfer in low-sample environments: Exploring meta-learning or few-shot learning strategies to mitigate the costs of large-scale data annotation and enhance generalization across diverse assembly scenarios with long-tail distributions.
- Semantic reasoning with knowledge graph integration: Integrating industrial knowledge graphs to transcend purely visual perception. By transforming process standards into structured knowledge chains, we aim to establish a “perception-cognition-decision” loop for deep semantic compliance verification.
- Generalization across the full assembly lifecycle: Extending the recognition framework from positioning and clamping to the full assembly lifecycle (e.g., drilling, riveting, gluing), advancing the industry toward fully digitalized and intelligent process supervision.

## Figures and Tables

**Table 1:** Statistical profile and class-wise duration of the LTSA-Dataset.

Action Category	Number of Video Clips	Total Duration/(min)	Average Duration/(s)
Rib datum marker	140	5.72	2.45
Rib positioning	126	9.18	4.37
Rib clamping1#	126	5.44	2.59
Rib clamping2#	126	5.19	2.47
Spar datum marker	154	4.83	1.88
Spar positioning	154	11.09	4.32
Spar clamping	154	15.79	6.15
Upper panel datum marker	140	7.14	3.06
Upper panel positioning	140	12.51	5.36
Upper panel clamp positioning1#	140	7.49	3.21
Upper panel clamp positioning2#	140	7.09	3.04
Upper panel clamp positioning3#	140	6.88	2.95
Upper panel clamping1#	140	14.75	6.32
Upper panel clamping2#	140	14.33	6.14
Upper panel clamping3#	140	14.58	6.25

**Table 2:** Table of compilation and runtime environment.

Compilation Environment	Settings Information
CPU	i9-12900K
GPU	NVIDIA GeForce RTX3090-24G
System memory	60 GB
Operating system	Windows10
Pytorch	1.10.1
CUDA + cuDNN	11.3 + 8.2.0.0

**Table 3:** Optimizer comparison experimental results table.

Optimizer	Top-1 Accuracy
C3D (SGD)	53.18%
C3D (Adam)	78.27%
C3D (AdamW)	81.12%

**Table 4:** Influence of the Mixup hyperparameter α on recognition accuracy (using AdamW).

Model	Top-1 Accuracy
C3D	81.12%
C3D + Mixup (α = 0.2)	84.05%
C3D + Mixup (α = 0.4)	83.64%
C3D + Mixup (α = 0.6)	82.79%

**Table 5:** Quantitative evaluation of BN layer integration.

Model	Top-1 Accuracy
C3D	84.05%
C3D + BN	89.83%

**Table 6:** Ablation results for different SimAM attention embedding strategies.

Model	Embedding Strategy	Top-1 Accuracy
C3D + BN	-	89.93%
C3D + SimAM1	After the pooling layers(five)	87.90%
C3D + SimAM2	Before the pooling layers(five)	90.76%
C3D + SimAM3	Before the pooling layers(eight)	93.02%

**Table 7:** Ablation results of LTSFEM placement across different convolutional layers.

Model	Conv1	Conv2	Conv3b	Conv4b	Top-1 Accuracy
C3D + BN + SimAM	-	-	-	-	93.02%
Conv1	✓	-	-	-	93.79%
Conv2	-	✓	-	-	95.08%
Conv3b	-	-	✓		95.29%
Conv4b	-	-	-	✓	94.88%
Conv2 + Conv3b	-	✓	✓		96.22%
Conv2 + Conv3b + Conv4b	-	✓	✓	✓	96.15%
Conv1 + Conv2 + Conv3b + Conv4b	✓	✓	✓	✓	94.79%

**Table 8:** Ablation results under varying structural configurations of the CWSTB module.

Model	Conv3a	Conv3b	Conv4a	Conv4b	Conv5a	Conv5b	Top-1 Accuracy
LTSA-nocwstb	-	-	-	-	-	-	96.22%
Conv5a + Conv5b	-	-	-	-	✓	✓	98.24%
Conv3a + Conv3b + Conv5a + Conv5b	✓	✓	-	-	✓	✓	97.96%
Conv4a + Conv4b + Conv5a + Conv5b	-	-	✓	✓	✓	✓	97.11%

**Table 9:** Module ablation comparison data table.

Model Configuration	Top1 Accuracy/%	Incremental
C3D	84.05	-
C3D + BN	89.93	+5.88%
C3D + BN + SimAM	93.02	+3.09%
C3D + BN + SimAM + LTSFEM	96.22	+3.20%
C3D + BN + SimAM + LTSFEM + CWSTB	98.24	+2.02%

**Table 10:** Detailed comparison results for all models.

Model	Accuracy/%	Parameters/M	Floating-Point Operations/GFLOPs	FPS
C3D	82.48	78.04	38.5	126
I3D	83.31	12.1	55.8	90
SlowFast	88.53	34.5	36.1	105
LTSA-Net	98.24	64.26	38.6	98
TimeSformer	91.25	121.4	59	32
Swin Transformer V2	94.60	28.2	23.5	41

**Table 11:** Classification performance on the LTSA-Net test Set.

Action Category	Precision/(%)	Recall/(%)	F1-Score/(%)
Class 0—Rib datum marker	93.75	100.00	96.77
Class 1—Rib positioning	100.00	83.33	90.91
Class 2—Rib clamping1#	93.33	93.33	93.33
Class 3—Rib clamping2#	93.33	96.55	94.92
Class 4—Spar datum marker	93.33	96.55	94.92
Class 5—Spar positioning	100.00	96.55	98.25
Class 6—Spar clamping	93.55	100.00	96.67
Class 7—Upper panel datum marker	100.00	82.76	90.57
Class 8—Upper panel positioning	82.35	96.55	88.89
Class 9—Upper panel clamp positioning1#	96.43	93.10	94.74
Class 10—Upper panel clamp positioning2#	90.00	93.10	91.53
Class 11—Upper panel clamp positioning3#	96.55	93.33	94.92
Class 12—Upper panel clamping1#	100.00	100.00	100.00
Class 13—Upper panel clamping2#	96.30	89.66	92.86
Class 14—Upper panel clamping3#	87.88	96.67	92.06

**Table 12:** Table of hardware configuration for real-world scenario validation.

	Front-Facing Camera A	Front-Facing Camera B
Type	MV-CS004-10UC	MV-CH050-10UC
Pixel size(um)	6.9	3.45
Physical dimensions	29 × 29 × 30	29 × 29 × 30
Resolution	720 × 540	2448 × 2048
Maximum frame rate	526.5 fps	74.1 fps
Lens mount/Interface	C	C
Data interface	USB 3.0	USB 3.0
Power supply	USB	USB

**Table 13:** Table of real-world scenario validation results.

Action Category	Total Occurrences	Correctly Recognized Occurrences	Recognition Accuracy
Rib datum marker	150	148	98.67%
Rib positioning	152	151	99.34%
Rib clamping1#	160	158	98.75%
Rib clamping2#	160	156	97.50%
Spar datum marker	148	147	99.32%
Spar positioning	150	150	100.00%
Spar clamping	152	151	99.34%
Upper panel datum marker	150	147	98.00%
Upper panel positioning	156	155	99.36%
Upper panel clamp positioning1#	148	146	98.65%
Upper panel clamp positioning2#	148	145	97.97%
Upper panel clamp positioning3#	148	146	97.33%
Upper panel clamping1#	150	150	100.00%
Upper panel clamping2#	150	149	99.33%
Upper panel clamping3#	150	148	98.67%
Total	2272	2247	98.82%

**Table 14:** Software recognition speed and real-time performance results.

Performance Metric	Test Result
Average processing frame rate	28 FPS
Average recognition time per frame	42 ms
Maximum processing time per frame	54 ms

## Data Availability

The original contributions presented in this study are included in the article. Further inquiries can be directed to the corresponding author.
